# Sleep Loss and Cytokines Levels in an Experimental Model of Psoriasis

**DOI:** 10.1371/journal.pone.0051183

**Published:** 2012-11-30

**Authors:** Camila Hirotsu, Mariana Rydlewski, Mariana Silva Araújo, Sergio Tufik, Monica Levy Andersen

**Affiliations:** 1 Departamento de Psicobiologia, Universidade Federal de Sao Paulo, Sao Paulo, Brazil; 2 Departamento de Bioquímica, Universidade Federal de Sao Paulo, Sao Paulo, Brazil; Northwestern University, United States of America

## Abstract

Up to 80% of people develop a cutaneous condition closely connected to their exposure to stressful life events. Psoriasis is a chronic recurrent inflammatory skin disorder with multifactorial etiology, including genetic background, environmental factors, and immune system disturbances with a strong cytokine component. Moreover, psoriasis is variably associated with sleep disturbance and sleep deprivation. This study evaluated the influence of sleep loss in the context of an animal model of psoriasis by measuring cytokine and stress-related hormone levels. Male adult Balb/C mice with or without psoriasis were subjected to 48 h of selective paradoxical sleep deprivation (PSD). Sleep deprivation potentiated the activities of kallikrein-5 and kallikrein-7 in the skin of psoriatic groups. Also, mice with psoriasis had significant increases in specific pro-inflammatory cytokines (IL-1β, IL-6 and IL-12) and decreases in the anti-inflammatory cytokine (IL-10) after PSD, which were normalized after 48 h of sleep rebound. Linear regression showed that IL-2, IL-6 and IL-12 levels predicted 66% of corticosterone levels, which were selectively increased in psoriasis mice subject to PSD. Kallikrein-5 was also correlated with pro-inflammatory cytokines, explaining 58% of IL-6 and IL-12 variability. These data suggest that sleep deprivation plays an important role in the exacerbation of psoriasis through modulation of the immune system in the epidermal barrier. Thus, sleep loss should be considered a risk factor for the development of psoriasis.

## Introduction

Psoriasis is a chronic inflammatory skin disease that affects 1–3% of the population [1]. Morphologically, psoriasis is characterized by epidermal hyperproliferation and neutrophil infiltrates in the epidermis. The accumulation of neutrophils appears to be related to the onset and maintenance of the acute phase of the disease, leading to skin hardening and flaking. Once present in the epidermis, the neutrophils release granules containing several enzymes, including active human neutrophil elastase (HNE) [2]. Keratinocyte proliferation is stimulated by the presence of HNE [3], which is found in abundance in the bottom membrane of psoriatic lesions [4]. In addition to HNE, other enzymes such as tryptases, metalloproteases and cathepsins B, L and D have also been linked to the pathogenesis and maintenance of psoriasis. These enzymes play an important role in keratinocyte proliferation [5].

Clinically, psoriasis is shown by sharply demarcated scaly erythematous plaques commonly found on the scalp, elbows, and knees. The disorder is thought to result from a polygenic predisposition [6] combined with triggering factors such as injury to the skin, infections [7], endocrine factors, and stress [8]. Strong evidence suggests that immune mechanisms, such as persistent activation of T-lymphocytes, excessive proliferation of keratinocytes, and reactivation of proto-oncogenes, may play a role in the pathogenesis of psoriasis [9]. Additionally, recent studies have demonstrated that cytokines can be found in the affected psoriatic areas and contribute significantly to the pathogenesis of the disease [10], [11], [12]. Moreover, the expression of kallikreins, mainly kallikrein-5 and kallikrein-7, is increased during the acute phase of psoriasis progression and is associated with abnormal differentiation of keratinocytes [13]. Kallikreins are major skin serine proteases responsible for early hydrolysis of corneodesmosomal proteins, such as desmoglein 1, desmocollin 1, and corneodesmosin, which leads to desquamation [14].

The impact of psoriasis on quality of life has been extensively investigated [15]. Psoriasis impairs the use of hands, walking, sitting, standing for long periods, sexual function, and sleep [16]. Particularly, poor sleep quality adversely affects quality of life in patients with psoriasis. Pruritus, depression, and pain interfere with sleep duration and structure by increasing nocturnal awakenings and leading to sleep deprivation and fragmentation [17]. Lack of sleep itself has important effects on immunological integrity and nocturnal secretion of cytokines [18], [19], [20], [21], [22], [23], [24] and may be considered another risk factor for psoriasis. This bi-directional interaction between the central nervous system and the immune system has been focus of intense research in recent decades [25]. In this sense, the current study aimed to understand the role sleep loss plays in psoriasis by examining related cytokine and hormonal profiles in an animal model. Understanding the contribution of sleep deprivation to psoriasis may help to improve the daily lives and psoriasis severity in patients by leading to novel therapeutic interventions.

## Materials and Methods

### Animals

The study was performed using 79 male Balb/C mice (20–30 g) from CEDEME (Centro de Desenvolvimento de Modelos Experimentais). For the first experiment (cytokines and corticosterone levels), a total of 49 animals were used [SHAM+CTRL (n = 10), SHAM+PSD (n = 10), PSO+CTRL (n = 11), PSO+PSD (n = 8) and PSO+SR48 (n = 10)]. For the second experiment (skin activity of kallikrein-5 and kallikrein-7), a total of 30 mice were used [SHAM+CTRL (n = 6), SHAM+PSD (n = 6), PSO+CTRL (n = 6), PSO+PSD (n = 6) and PSO+SR48 (n = 6)]. The animals were maintained under controlled conditions with a light-dark period of 12∶12 h, temperature of 22±1°C, and free access to a commercial diet (Nuvilab) and water. The animals had a 2 week adaptation period following transfer from CEDEME, after which they were placed in individual standard cages. All procedures used in the present study complied with the National Institutes of Health Guide for Care and Use of Laboratory Animals. All animal procedures were approved by the Ethical Committee of the Universidade Federal de Sao Paulo (#0233/08).

### Experimental protocol

**Figure 1:**
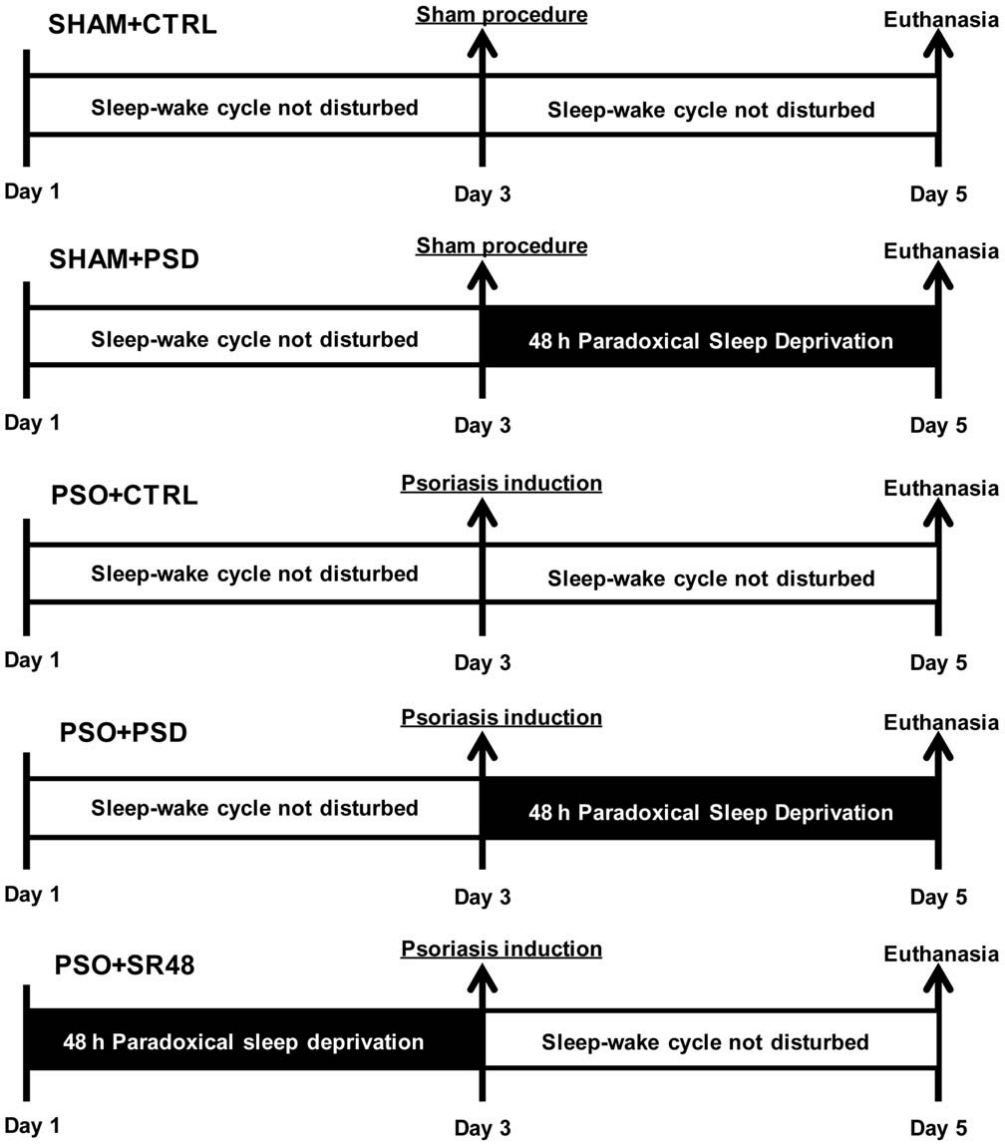
Experimental protocol. Experimental timeline for each condition: SHAM+CTRL (sham procedure followed by sleep control condition, n = 10), SHAM+PSD (sham procedure followed by 48 h of paradoxical sleep deprivation, n = 10), PSO+CTRL (psoriasis induction followed by sleep control condition, n = 11), PSO+PSD (psoriasis induction followed by 48 h of paradoxical sleep deprivation, n = 8) and PSO+SR48 (psoriasis induction followed by 48 h of sleep rebound, n = 10).

Animals were randomly assigned to SHAM (sham procedure) or PSO (psoriasis induction) group. These groups were further divided into either the control (CTRL) or paradoxical sleep deprivation (PSD) conditions. The animals in CTRL condition were kept undisturbed in their home cages with normal access to sleep-wake cycle (regular 12∶12 h light/dark cycle). However, the animals in PSD condition had their sleep-wake cycle disturbed by 48 h of sleep deprivation, as further described below. Finally, another group received PSD followed by 48 h of sleep rebound (free opportunity for sleep recovery in the home cage). Figure 1 shows the experimental design with 5 groups: SHAM+CTRL (sham procedure in control conditions), SHAM+PSD (sham procedure followed by 48 h of paradoxical sleep deprivation), PSO+CTRL (psoriasis induction in control conditions), PSO+PSD (psoriasis induction followed by 48 h of paradoxical sleep deprivation) and PSO+SR48 (48 h of paradoxical sleep deprivation followed by psoriasis induction and 48 h of sleep rebound). We elected to not include a SHAM+SR48 group since our main objective was to evaluate whether the changes caused by sleep deprivation in the psoriasis model would be recovered by 48 h of sleep rebound.

### Induction of psoriasis

Psoriasis induction was induced by HNE protocol [3]. This model was chosen since it causes a number of structural and functional abnormalities characteristic of early psoriasis stages with clear pathogenesis, in contrast to other models of spontaneous mutations (asebia, chronic proliferative dermatitis, and flaky skin) which lack clear pathogenesis [26], [27], [28]. Also, the concentrations of HNE tested in the HNE model are comparable with those found on the skin surface of psoriatic lesions [29], and it better mimics the early stages of psoriasis as HNE is detected predominantly in acute lesions in humans [4]. However, the HNE model has not yet been evaluated for its contribution for the immunopathogenesis of psoriasis. Although many other animal models appear to be more suitable to examine the role of cytokines because of their immunological basis (K14/IL1α, K14/IL-6, involucrin/IFNγ, H14/TGF/alpha, K10/BMP-6 models) [30], [31], [32], [33], [34], it seems that a single cytokine alone is not sufficient to induce the complex tissue alterations that characterize psoriasis; it is more likely that psoriasis involves a cytokine network [35]. Also, all of these transgenic rodent models present relatively low penetrance of the psoriasiform phenotypes.

Briefly, on the third day of the experimental protocol (Figure 1), all the animals were anesthetized with ketamine and xylazine (10 mg/kg and 5 mg/kg body weight, respectively, i.p.) and had the dorsal region trichotomized. The skin was treated with acetone and a 1 cm^2^ area marked, which was then covered with an adherent aluminum chamber (Finn Chambers on Scanpor, Hermal, Reinbek, Germany). In animals assigned to PSO induction, the skin beneath the chamber was then treated with a solution of HNE 10 nM pH 7.5 (20 µL) (Merck, Brazil). The SHAM groups received vehicle buffer Tris 50 mM pH 7.5 (20 µL) on the skin covered by the adherent aluminum chamber. Body weight was measured on day 1 and day 5 of the experimental protocol.

### Paradoxical sleep deprivation

Animals in the PSD condition were paradoxically sleep deprived for 48 h using the modified multiple platform method. To minimize the stress associated with the PSD, the animals were adapted to this method during 3 consecutive days for a short period of time. The PSD started before (PSO+SR48 group) and after (SHAM+PSD and PSO+PSD groups) psoriasis induction or sham procedure. Each mouse was individually placed inside the cage (34×18×12 cm) containing 5 circular platforms (3.5 cm in diameter) with water up to 1 cm of their upper surface. At the onset of each paradoxical sleep episode, the animals present a loss of muscle tonus, causing them to fall into the water and be awakened. Previous experiments have shown that this method causes a total suppression of paradoxical sleep in mice and a mild reduction of slow wave sleep [36], [37]. Food and water were available *ad libitum* during PSD and the water in the cage was changed once a day. For the CTRL condition and the sleep rebound period of PSO+SR48, animals were kept individually in their home cages (undisturbed sleep-wake cycle in normal bedding with free access to water and food) in the same room as the PSD groups. We did not include large platform controls, which is a stress control method widely accepted in sleep research, since we elect to control for this effect by measuring corticosterone levels. Large platforms controls present disturbed sleep architecture that could interfere in this study [38].

### Blood samples for biochemical and hormonal evaluation

Forty-eight hours after PSD or sleep recovery, animals were euthanized by rapid decapitation. Blood was collected in sterile tubes containing liquid EDTA and centrifuged (4000 rpm at 4°C) to obtain samples of plasma. Samples were stored at −80°C until the assays. Cytokine determinations were performed on duplicate plasma samples using the FlowMetrix System (Luminex100™ platform), a multiplexed fluorescent bead-based immunoassay which permits the simultaneous quantitation of multiple cytokines. A custom 8-plex kit specific for mouse cytokines (Millipore, San Francisco, CA, USA) was used to analyze interleukin (IL)-1β, IL-2, IL-6, IL-10, IL-12, IL-17, interferon γ (IFNγ) and tumor necrosis factor α (TNFα) in each 50-µl plasma sample. Sensitivity of the assay was 2 pg/ml for each cytokine; all samples were run in the same assay to avoid interassay variability. Concentrations of the stress-related hormone, corticosterone, were assayed by a double antibody radioimmunoassay method using a commercial kit specific for mice (MP Biomedicals, NY, USA). The sensitivity of the assay was 0.25 ng/ml.

### Kallikrein activity

Skin samples from each experimental group were processed in buffer (Tris 50 mM, NaCl 500 mM, Tween 20 0.005%, pH 8.0) for the kallikrein activity assays. Kallikrein-like activity was assayed by the use of Abz-KLRFSKQ-EDDnp (for kallikrein-5; k5) and Abz-KLRSSKQ-EDDnp (for kallikrein-7; k7). For k5-like activity, samples were incubated with 50.0 µM PPACK II for 10 min at 37.0°C and then 10.0 µM of the substrate was added. For k7-like activity, samples were incubated with 50.0 µM PPACK II or 100 µM TPCK for 10 min at 37.0°C and then 10.0 µM of the substrate was added. Control reactions were held in the absence of the inhibitors. Hydrolysis of the substrates was measured in λ = 320 nm λ = 420 nm in the microplate reader.

### Statistical methods

All data met the assumptions of normality and homogeneity, and so were evaluated using one-way ANOVAs followed by post-hoc Tukey's test, when necessary. For body weight analysis, one-way ANOVA with repeated measures was used, followed by Tukey's test. To evaluate the relationship between cytokines and corticosterone levels and cytokines and kallikrein activity, a Pearson correlation test was performed. Cytokines that presented a significant correlation with the dependent variable were included in a multiple linear regression model using the stepwise way. As the data were collected from 2 repetitions of the experimental protocol, correlation and regression tests were performed with data obtained from the second experiment (n = 6/group), in which skin tissue was collected for kallikrein activity as well as blood was collected for additional cytokine and corticosterone quantification. The data are expressed as mean ± SEM. The level of significance was set at p≤0.05. All analyses were performed using SPSS 17 (SPSS Inc., Chicago, IL, USA).

## Results

**Figure 2:**
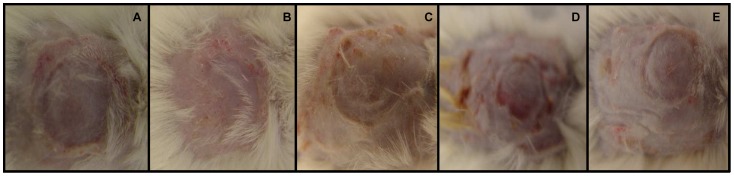
Skin illustration. Representative pictures of skin from different mice groups after the experimental protocol: SHAM+CTRL (**A**), SHAM+PSD (**B**), PSO+CTRL (**C**), PSO+PSD (**D**) and PSO+SR48 (**E**).

**Figure 3:**
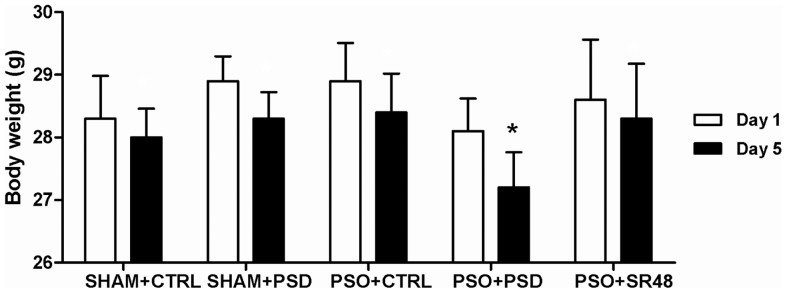
Body weight. Initial (Day 1) and final (Day 5) body weight in different groups: SHAM+CTRL (sham procedure followed by sleep control condition, n = 10), SHAM+PSD (sham procedure followed by 48 h of paradoxical sleep deprivation, n = 10), PSO+CTRL (psoriasis induction followed by sleep control condition, n = 11), PSO+PSD (psoriasis induction followed by 48 h of paradoxical sleep deprivation, n = 8) and PSO+SR48 (psoriasis induction followed by 48 h of sleep rebound, n = 10). *p<0.05 compared to respective initial body weight. Values expressed as mean ± SEM.

**Figure 4:**
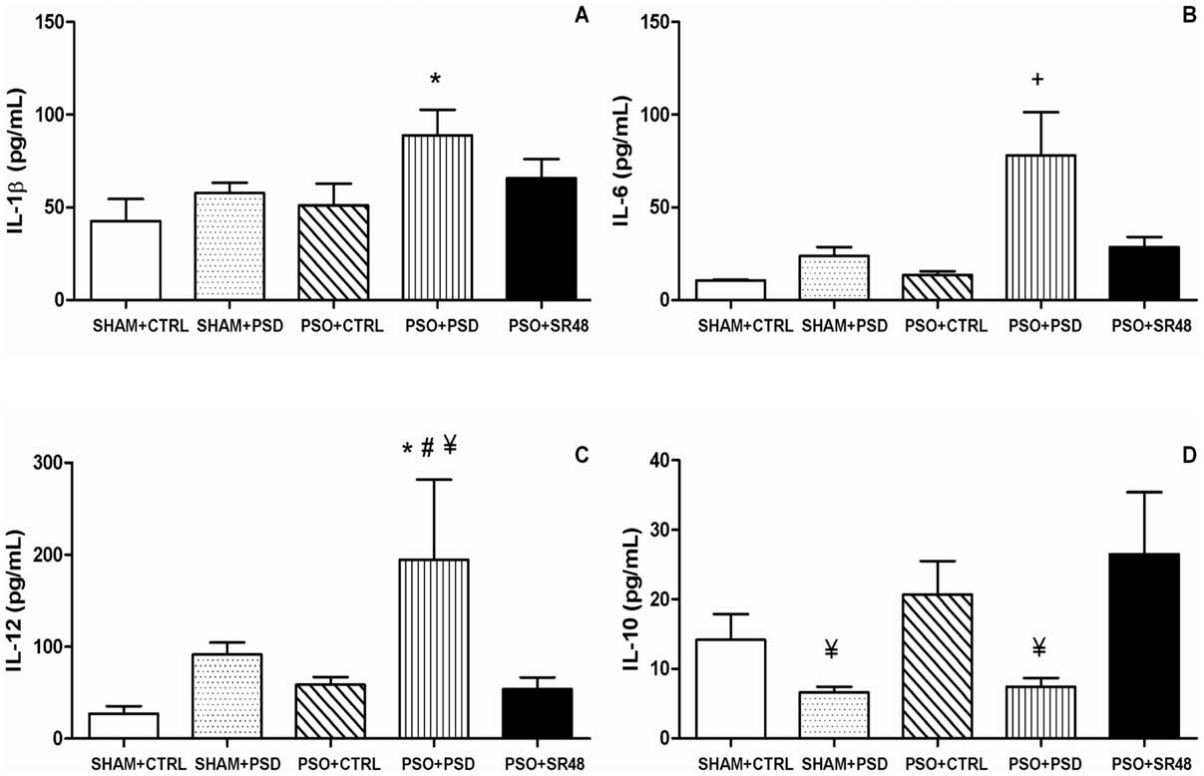
Circulating cytokines. Plasma concentration of cytokines **A**) IL-1β (pg/mL), **B**) IL-6 (pg/mL), **C**) IL-12 (pg/mL) and **D**) IL-10 (pg/mL) in different groups: SHAM+CTRL (sham procedure followed by sleep control condition, n = 10), SHAM+PSD (sham procedure followed by 48 h of paradoxical sleep deprivation, n = 10), PSO+CTRL (psoriasis induction followed by sleep control condition, n = 11), PSO+PSD (psoriasis induction followed by 48 h of paradoxical sleep deprivation, n = 8) and PSO+SR48 (psoriasis induction followed by 48 h of sleep rebound, n = 10). *p<0.05 compared to SHAM+CTRL; #p<0.05 compared to PSO+CTRL; ¥p<0.05 compared to PSO+SR48; +p<0.05 compared to all groups. Values expressed as mean ± SEM.

The induction protocol reliably resulted in psoriasis by day 5 (Figure 2). Body weight was only significantly decreased across the course of the experiment in PSO+PSD group (*F*(4,44) = 7.03, *p*<0.05; Figure 3). In relation to the circulating cytokines levels, ANOVA revealed differences between the groups for the following interleukins: IL-1β (*F*(4,44) = 2.37, *p*<0.05), IL-6 (*F*(4,44) = 6.17, *p*<0.05), IL-10 (*F*(4,44) = 2.32, *p*<0.05) and IL-12 (*F*(4,44) = 3.43, *p*<0.05) (Figure 4). No statistical differences were observed for IFNγ, IL-2, TNFα and IL-17 (*p*>0.05) (Table 1). Subsequent post-hoc tests demonstrated that IL-1α was increased in the PSO+SR48 group compared to all other groups (*p*<0.05); IL-1β showed an increase in the PSO+PSD group compared to SHAM+CTRL (*p*<0.05); IL-6 was higher in the PSO+PSD group compared to all other groups (*p*<0.05); and IL-12 was significantly increased in the PSO+PSD group compared to SHAM+CTRL, PSO+CTRL and PSO+SR48 groups (*p*<0.05).

**Table 1:** Cytokines.

Group	IFNγ (pg/mL)	IL-2 (pg/mL)	TNFα (pg/mL)	IL-17 (pg/mL)
**SHAM+CTRL**	14.7±1.6	90.7±14.2	15.2±1.2	3.1±2.1
**SHAM+PSD**	15.5±2.0	122.8±17.7	16.2±0.5	5.8±0.8
**PSO+CTRL**	15.0±2.4	85.1±16.2	16.2±1.3	4.8±1.2
**PSO+PSD**	17.4±2.5	127.5±9.7	21.6±6.3	6.9±2.3
**PSO+SR48**	14.9±2.8	94.1±15.7	20.2±2.3	9.2±5.0

Plasma concentration of cytokines IFNγ (pg/mL), IL-2 (pg/mL), TNFα (pg/mL) and IL-17 (pg/mL) in different groups: SHAM+CTRL (sham procedure followed by sleep control condition, n = 10), SHAM+PSD (sham procedure followed by 48 h of paradoxical sleep deprivation, n = 10), PSO+CTRL (psoriasis induction followed by sleep control condition, n = 11), PSO+PSD (psoriasis induction followed by 48 h of paradoxical sleep deprivation, n = 8) and PSO+SR48 (psoriasis induction followed by 48 h of sleep rebound, n = 10). No statistically significant differences. Values expressed as mean ± SEM.

**Figure 5:**
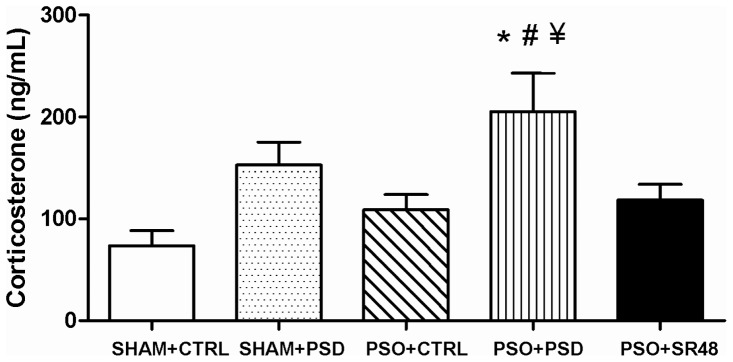
Stress hormone. Plasma concentration of corticosterone (ng/mL) in different groups: SHAM+CTRL (sham procedure followed by sleep control condition, n = 10), SHAM+PSD (sham procedure followed by 48 h of paradoxical sleep deprivation, n = 10), PSO+CTRL (psoriasis induction followed by sleep control condition, n = 11), PSO+PSD (psoriasis induction followed by 48 h of paradoxical sleep deprivation, n = 8) and PSO+SR48 (psoriasis induction followed by 48 h of sleep rebound, n = 10). *p<0.05 compared to SHAM+CTRL; #p<0.05 compared to PSO+CTRL; ¥p<0.05 compared to PSO+SR48; +p<0.05 compared to all groups. Values expressed as mean ± SEM.

ANOVA revealed differences in corticosterone levels between groups (*F*(4,44) = 4.43, *p*<0.05), with post hoc tests showing that corticosterone was increased in the PSO+PSD group compared to SHAM+CTRL, PSO+CTRL and PSO+SR48 groups (*p*<0.05) (Figure 5). Pearson's correlation analysis between each cytokine and corticosterone levels revealed small positive correlations for IFNγ (*R* = 0.36, *p*<0.05), IL-2 (*R* = 0.49, *p*<0.001), IL-6 (*R* = 0.40, *p*<0.05) and IL-12 (*R* = 0.42, *p*<0.01; Table 2). Multiple linear regression performed with IFNγ, IL-2, IL-6 and IL-12 as predictors of corticosterone levels demonstrated that IL-2 (*Beta* = 0.53, *t* = 3.85, *p*<0.001), IL-6 (*Beta* = 0.46, *t* = 3.34, *p*<0.01) and IL-12 (*Beta* = 0.39, *t* = 2.84, *p*<0.01) entered the model (*R* = 0.81; *R* ^2^ = 0.66; *p*<0.0001) (Table 3).

**Table 2:** Association between cytokines and cortiscosterone.

	*R*	*p*
**IFNγ (pg/mL)**	0.36	**0.02**
**IL-1α (pg/mL)**	−0.08	0.62
**IL-1β (pg/mL)**	0.24	0.13
**IL-2 (pg/mL)**	0.49	**0.001**
**IL-6 (pg/mL)**	0.40	**0.02**
**TNFα (pg/mL)**	0.21	0.18
**IL-10 (pg/mL)**	−0.16	0.36
**IL-12 (pg/mL)**	0.42	**0.01**
**IL-17 (pg/mL)**	0.09	0.59

Univariate analysis of factors associated with corticosterone levels (Pearson correlation test, N = 30).

**Table 3:** Corticosterone predictors.

	*Beta*	*t*	*p*
**(Constant)**		−1.33	0.20
**IL-2 (pg/mL)**	0.53	3.85	**0.001**
**IL-6 (pg/mL)**	0.46	3.34	**0.01**
**IL-12 (pg/mL)**	0.39	2.84	**0.01**

Multivariate analysis with corticosterone as the dependent variable (Multiple linear regression, N = 30).

**Figure 6:**
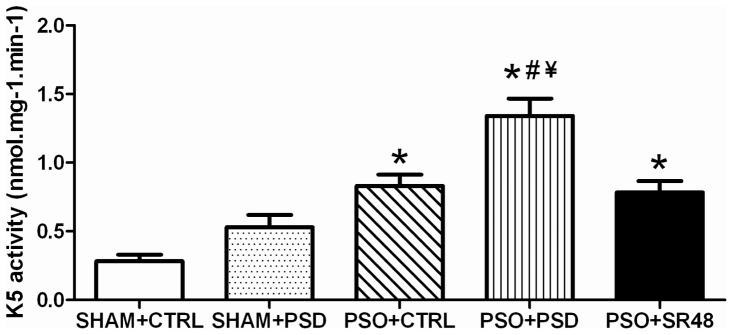
Kallikrein-5. Skin kallikrein-5 (K5) activity (nmol.mg^−1^.min^−1^) in different groups: SHAM+CTRL (sham procedure followed by sleep control condition, n = 6), SHAM+PSD (sham procedure followed by 48 h of paradoxical sleep deprivation, n = 6), PSO+CTRL (psoriasis induction followed by sleep control condition, n = 6), PSO+PSD (psoriasis induction followed by 48 h of paradoxical sleep deprivation, n = 6) and PSO+SR48 (psoriasis induction followed by 48 h of sleep rebound, n = 6). *p<0.01 compared to SHAM+CTRL; #p<0.001 compared to SHAM+PSD; ¥p<0.05 compared to PSO+CTRL and PSO+SR48. Values expressed as mean ± SEM.

**Figure 7:**
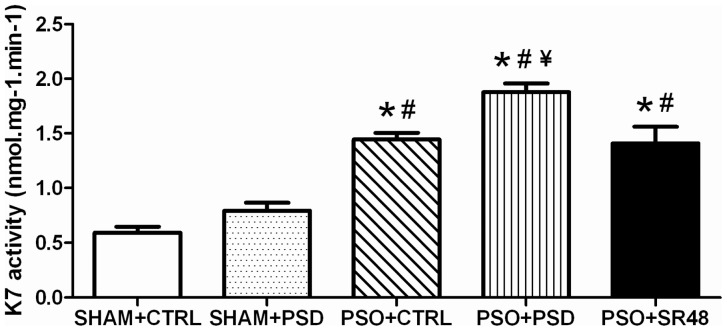
Kallikrein-7. Skin kallikrein-7 (K7) activity (nmol.mg^−1^.min^−1^) in different groups: SHAM+CTRL (sham procedure followed by sleep control condition, n = 6), SHAM+PSD (sham procedure followed by 48 h of paradoxical sleep deprivation, n = 6), PSO+CTRL (psoriasis induction followed by sleep control condition, n = 6), PSO+PSD (psoriasis induction followed by 48 h of paradoxical sleep deprivation, n = 6) and PSO+SR48 (psoriasis induction followed by 48 h of sleep rebound, n = 6). *p<0.01 compared to SHAM+CTRL; #p<0.001 compared to SHAM+PSD; ¥p<0.05 compared to PSO+CTRL and PSO+SR48. Values expressed as mean ± SEM.

For the kallikrein-5 activity, ANOVA showed a group effect (*F*(4,25) = 19.59, *p*<0.0001) with increased activity in PSO+CTRL, PSO+PSD and PSO+SR48 groups compared to SHAM+CTRL (*p*<0.01) (Figure 6). Moreover, PSO+PSD group had increased kallikrein-5 activity compared with SHAM+PSD, PSO+CTRL and PSO+SR48 groups (*p*<0.01). Similarly, kallikrein-7 activity had differences between the groups (*F*(4,25) = 33.05, *p*<0.0001), showing increased activity in PSO+CTRL, PSO+PSD and PSO+SR48 compared to SHAM+CTRL and SHAM+PSD groups (*p*<0.001) (Figure 7). PSO+PSD potentiated the increase in kallikrein-7 activity in relation to PSO+CTRL and PSO+SR48 groups (*p*<0.05). Pearson's correlation analysis between cytokines, corticosterone and kallikrein-5 showed a significant association between kallikrein-5 activity and the variables IL-6 (*R* = 0.59, *p*<0.01), IL-12 (*R* = 0.50, *p*<0.01), IL-17 (*R* = 0.52, *p*<0.01), and corticosterone (*R* = 0.47, *p*<0.05) (Table 4). In relation to kallikrein-7, the correlation was significant only for IL-12 (*R* = 0.46, *p*<0.01) (Table 4). Multiple linear regression performed with IL-6, IL-12, IL-17 and corticosterone as predictors of kallikrein-5 activity demonstrated that only the pro-inflammatory cytokines IL-6 (*Beta* = 0.59, *t* = 3.94, *p*<0.001) and IL-12 (*Beta* = 0.46, *t* = 3.08, *p*<0.01) entered the model (*R* = 0.76; *R* ^2^ = 0.58; *p*<0.0001) (Table 5).

**Table 4:** Association between kallikreins, cytokines and corticosterone.

		IFNγ	IL-1β	IL-2	TNFα	IL-12	IL-6	IL-10	IL17	Corticosterone
**Kallikrein-5**	***R***	0.22	0.35	0.17	0.33	0.50	0.59	0.14	0.52	0.47
	***p***	0.22	0.06	0.37	0.08	**0.01**	**0.01**	0.51	**0.01**	**0.02**
**Kallikrein-7**	***R***	0.04	0.17	0.08	0.31	0.46	0.34	0.05	0.30	0.37
	***p***	0.82	0.38	0.69	0.10	**0.01**	0.10	0.80	0.14	0.07

Univariate analysis of factors associated with kallikrein-5 and kallikrein-7 activities (Pearson correlation test, N = 30).

**Table 5:** Kallikrein-5 predictors.

	*Beta*	*t*	*p*
**(Constant)**		6.17	**0.0001**
**IL-6 (pg/mL)**	0.59	3.94	**0.001**
**IL-12 (pg/mL)**	0.46	3.08	**0.006**

Multivariate analysis with kallikrein-5 as the dependent variable (Multiple linear regression, N = 30).

## Discussion

The current study examined the effects of sleep deprivation on plasma cytokines in an experimental model of psoriasis. As expected, all psoriatic animals had increases in kallikrein-5 and kallikrein-7 activity, which were potentiated by sleep deprivation, and attenuated by 48 h of sleep rebound. Also, pro-inflammatory cytokines (IL-1β, IL-6 and IL-12) were significantly increased solely in the PSO+PSD group, and were restored to normal levels after 48 h of sleep rebound. However, a sleep deprivation-dependent decrease in the anti-inflammatory cytokine (IL-10) was observed in both SHAM+PSD and PSO+PSD, suggesting sleep deprivation may be an important risk condition for psoriasis [39]. In addition, a multiple linear regression model revealed an association between the activity of the kallikreins and pro-inflammatory cytokines, especially IL-6 and IL-12, which significantly predicted kallikrein-5 activity. The same pattern was observed in corticosterone concentrations, which were elevated only in psoriasis mice subjected to PSD. These changes were also directly correlated to the immune response and to skin kallikrein activity in psoriasis mice. Together, the results suggest a relationship between hypothalamus-pituitary-adrenal (HPA) axis activation and pro-inflammatory/anti-inflammatory cytokine imbalance in psoriasis animals challenged with sleep deprivation.

Stress is a relevant factor in triggering psoriasis symptomatology [40], [41] and is intrinsically related to sleep deprivation through HPA activation [42], [43], [44]. Evidence suggests that the efficacy of corticosterone-mediated feedback determines the characteristics of basal and stress-related activity [45]. Importantly, the current study found a positive correlation between corticosterone and pro-inflammatory cytokines, which were significantly increased in the PSO+PSD group. Remarkably, the increase in IL-2, L-6, and IL-12 were responsible for at least 66% of the changes observed in corticosterone levels. This result indicates that PSD is associated with changes in immune function which are known to be involved in psoriasis. This may be one potential explanation of how stress triggers psoriatic eruption.

Our results also demonstrate a cause-effect relationship between the immune response and kallikrein-5 activity, showing that the pro-inflammatory cytokines IL-2 and IL-12 explain 58% of the changes observed in kallikrein-5 activity. These data suggest that sleep deprivation may change the cytokine environment leading to excessive protease activity, which in turn affects the epidermal barrier and contributes to psoriasis pathogenesis. However, this may not be directly dependent on the HPA axis activation, since corticosterone was not a predictor of kallikrein-5 activity, but only associated with it. On the other hand, Morizane et al. [46] showed no changes in kallikrein-5 expression of normal human epidermal keratinocytes stimulated with type-2 cytokines (IL-4 and IL-13). Although this was an *in vitro* study, the authors found that only type-2 cytokines were able to increase kallikrein-7 expression and function in an atopic dermatitis model [46].

Our data also demonstrated that the PSD and psoriasis induction themselves were not enough to cause an HPA stress-related response or to change the circulating cytokines profile, although the combination of the PSD and psoriasis presented synergistic effects. However, the PSO+SR48 group showed that the reestablishment of sleep homeostasis was essential to restore the balance between pro-inflammatory/anti-inflammatory cytokines and corticosterone levels. In patients, it is difficult to isolate the stress related to lifestyle from the pathophysiology of the disease; these results could explain at least in part the contradiction found between the neuroendocrine profile of the HPA axis and cytokines in psoriatic patients [47], [48], i.e., the level of cytokines could change given a certain level of cortisol in psoriasis patients. However, in contrast to previous studies [49], [50] we did not find increased levels of type-1 cytokines such as IFNγ, TNFα, IL-6 and IL-12 or decreases in type-2 cytokines like IL-10 [12], [51] in PSO mice that were not sleep deprived. Only mice in the PSO+PSD developed significant alterations in the cytokine network. This may be explained by the short period established for the duration of the psoriasis model. But, despite the short induction of psoriasis, the PSD challenge was still able to induce inflammatory markers, which suggests that the effects of PSD can be severe.

Cytokine imbalance represents an interesting target in psoriasis. Overexpression of IL-6 and IL-12 has been implicated in the pathology of psoriasis in addition to several other autoimmune and chronic inflammatory diseases [11], [52], [53], [54]. IL-6 induces an acute inflammatory reaction and supports the activation of lymphocytes, myeloid cells and keratinocytes in the epidermis, which may increase the serum level of IL-6, leading to increased inflammation. Relative deficiency of IL-10 is also seen in psoriatic patients and may be important in the development of this disease [55], [56], [57]. However, no significant differences were found in the polymorphisms of IL-6 and IL-10 promoter genes between patients with psoriasis and healthy controls [58]. Of note, PSD has been demonstrated to increase type-1 cytokines IL-1β, IL-6 and IL-12 levels in rats in a sleep loss-dependent manner [59], [60]. Since our results show that these cytokines can modulate the kallikrein-5 function, it is important to highlight the importance of good sleep quality for maintenance of skin integrity.

It is important to note that the main cytokine alterations were observed only with the combination of both sleep deprivation and psoriasis. This may be due to the animal model chosen for this study. Another animal model based on T-cell transfers to pathogen-free scid/scid mice (CD4+/CD45RB^hi^ model) presents an inflammatory cytokine response via dysregulated T-cells with altered keratinocyte differentiation, mimicking human psoriasis [61]. It takes, however, around 8 weeks to be completely established in all subjects and also causes intestinal inflammation in addition to the skin inflammation [62], [63]. As our main goal was to evaluate the effects of the interaction between sleep deprivation and early stages of psoriasis on cytokines levels, we chose the HNE model due to concerns that possible cytokine changes could be masked in the CD4+/CD45RB^hi^ model due to high baseline cytokine levels. However, additional studies in this model should be performed to expand upon and generalize our results. Clearly, there is no perfect model for psoriasis. Each one is based on a different mechanism with its similarities to psoriasis as well as its limitations.

Circadian variations have been found in cytokines levels [64], [65], [66]. Many facets of inflammation such as pro-inflammatory cytokines present circadian pattern in humans, typically peaking during the night [67]. People suffering from rheumatoid arthritis exhibit diurnal variation in disease severity associated with fluctuations in IL-6 levels [68]. Also, significant temporal dependence of LPS-induced shock is reported in mice [67], [69] and this response is potentiated by circadian disruption mimicking jet lag [70]. Increasing evidence also shows that the circadian rhythm, in comparison to sleep deprivation, favors the production of type-1 cytokines [71]. Immune factors such as cytokines can also influence the phase of the circadian clock, providing a bidirectional flow of circadian information in the immune system. For example, the circadian clock protein Per 2 has been shown to control the expression of the pattern recognition receptor TLR9, and this regulation translates to daily variations in TLR9 responsiveness modulated by cytokine response [72]. Although important, it is ultimately difficult to dissect the influence of sleep from the biological clock. The marked sleep-wake rhythm is controlled by both the circadian system and sleep, but also by confounding influences like body posture, physical activity, feeding and fasting, lighting, and ambient temperature [73]. Thus, considering the interference of the circadian rhythm may be difficult in the current study. Since the effect of circadian rhythm vs. sleep deprivation clearly depends on the time of the assessment, a single blood draw cannot provide conclusive data. Some evidence shows that psoriatic patients present disturbed circadian rhythms due to a loss of the nocturnal peak in melatonin secretion [74]. In addition, psoriasis patients show desynchronized circadian acrophases compared to control subjects in many other variables such as oral temperature, arterial blood pressure, pulse, and electrolytes [75]. Taken together, these data suggest that additional studies on the circadian rhythm should also be considered for a better understanding of the interactions between sleep deprivation and cytokines in the development of psoriasis.

This is the first study to investigate the involvement of sleep deprivation in the inflammatory and stress-related response of an animal model of psoriasis. Our results add supporting evidence to novel therapeutic options (such as anti-interleukins treatments and sleep quality care with polysomnography monitoring) as well as a better understanding of the role of sleep in the pathophysiology of inflammatory skin diseases such as psoriasis. Moreover, the positive correlation of a stress-related hormone with circulating type-1 cytokines and kallikrein activity suggested that altered HPA activity can be a consequence of the immune response associated with the interaction between psoriasis and sleep deprivation.

Future studies should examine longer durations for the psoriasis model as well as histological skin analysis that could be correlated with the systemic cytokine response. Also, sleep recordings would be important to characterize the sleep pattern in this psoriasis model in order to verify the distribution of sleep stages and their relationship with the cytokine profile. Although in humans, acute sleep deprivation and chronic sleep restriction are more commonly observed than PSD, the relevance of this method to the human condition is addressed in the mechanisms it can clearly provide, showing the importance of a specific sleep stage (paradoxical sleep or REM) in the context of psoriasis. In conclusion, our data suggest that sleep plays an important role in the exacerbation of psoriasis via modulation of the immune system, and that sleep loss should be considered a risk factor for the initiation, development, and recurrence of psoriasis.
